# Transient hypogammaglobulinemia of infancy may be associated with reduced switched memory B cells and del (16) (p11.2p12)

**DOI:** 10.1002/ccr3.3837

**Published:** 2021-06-22

**Authors:** Tsuyoshi Ito, Shotaro Iwamoto, Masahiro Hirayama, Yasuharu Yamada, Eiichi Azuma

**Affiliations:** ^1^ Department of Pediatrics Toyohashi Municipal Hospital Toyohashi Japan; ^2^ Department of Pediatrics Mie University Graduate School of Medicine Tsu Japan; ^3^ Department of Clinical Engineering Suzuka University of Medical Science Suzuka Japan

**Keywords:** chromosomal abnormality, common variable immunodeficiency, switched memory B cell, transient hypogammaglobulinemia of infancy

## Abstract

Transient hypogammaglobulinemia of infancy may be associated with chromosome del (16)(p11.2) that has reportedly been associated with other forms of primary immunodeficiency (*Clin Immunol*, 2009, 131, 24; *J Allergy Clin Immunol*, 2015;135, 1569).

## Graphical abstract

Transient hypogammaglobulinemia of infancy may be associated with chromosome del (16)(p11.2) that has reportedly been associated with other forms of primary immunodeficiency^1,2^.

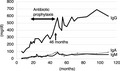

## 1 INTRODUCTION

Transient hypogammaglobulinemia of infancy (THI) is classically described as prolongation of the "physiologic" immunoglobulin nadir that is normally observed during the first three to six months of life. Immunoglobulin G (IgG) levels usually recover by the age of 2 to 4 years and may recover in adolescence and adulthood in some patients.^1^ The underlying basis for this disorder is unknown and may be heterogeneous. Diagnosis is also made retrospectively upon normalization of immunoglobulin levels. Infants with THI must be distinguished from those with permanent forms of primary immunodeficiency such as common variable immunodeficiency disorders (CVID), in which switched memory B cells are diminished or absent and are considered a good biomarker in CVID patients.^2^ Flow cytometric analysis of class‐switched memory B cells in the peripheral blood of CVID patients can also reveal defects at various stages of B‐cell differentiation in CVID subgroups.^2^

We encountered an infant with hypogammaglobulinemia who also had developmental delay and was finally diagnosed as having THI at 5 years of age. Interestingly, he also had a genetic abnormality, namely del (16) (p11.2p12) that has not been reported to be associated with THI so far.

16p11.2 deletion syndrome, Online Mendelian Inheritance in Man (OMIM) #611913, is a genetic disorder with varying estimates of prevalence from as low as 3:10 000^3^ to as common as 1:235.^4^ Individuals with 16p11.2 deletion syndrome usually have developmental delay, intellectual disability, and minor physical abnormalities such as low‐set ears or partial syndactyly. Other individuals with the 16p11.2 deletion have no associated health or behavioral problems, and so the deletion may never be detected. Maggadottir reported rare variants at 16p11.2 were associated with CVID.^5^ Here, we discuss the possibility that THI may be associated with reduced switched memory B cells and del (16) (p11.2p12) in the same patient.

## 2 CASE PRESENTATION

**FIGURE 1:**
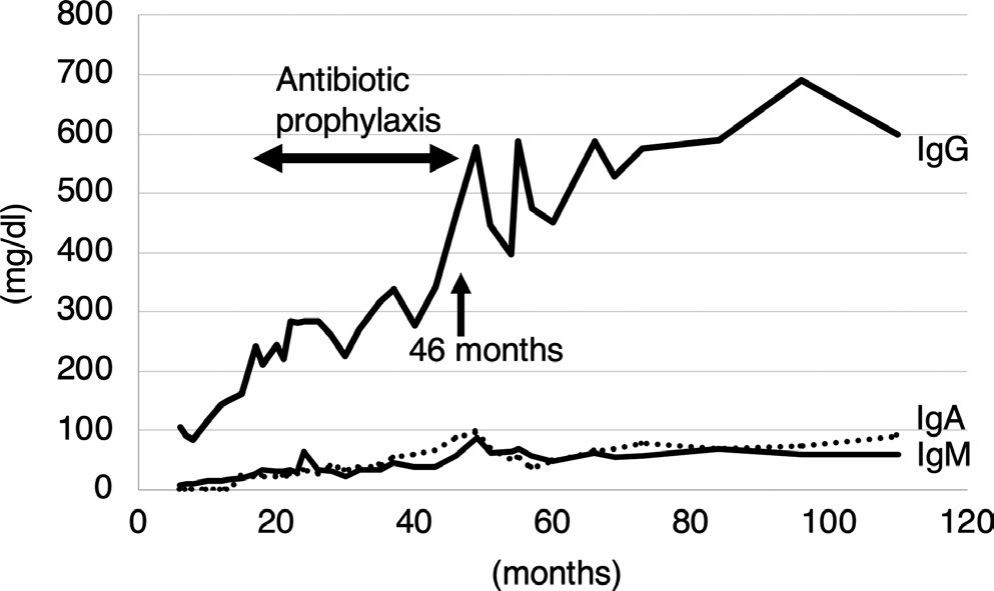
Time course of immunoglobulin levels. IgG levels fluctuated during 4 to 5 years of age, then normalized thereafter. IgA and IgM were gradually increasing

A 10‐year‐old boy was born at 38 gestational weeks with 1759g of birth weight and favorable Apgar score. He did not have other abnormalities on physical examination. His mother had been diagnosed with antiphospholipid syndrome. Hypogammaglobulinemia was detected when he developed axillary lymphadenitis following BCG vaccination at 6 months of age. It was successfully treated with a beta‐lactam antibiotic. Immunologic evaluation revealed hypogammaglobulinemia‐IgG 105 mg/dL (5‐12 month reference range: 172‐1069 mg/dL), IgA < 10 mg/dL (4‐6 month reference range: 4.4‐84 mg/dL), and IgM 7 mg/dL (5‐9 month reference range: 33‐126 mg/dL).^6^ He did not have secondary causes of hypogammaglobulinemia such as corticosteroid therapy or protein‐losing states. The frequency of total B cells within the lymphocytes was 17%, which was within normal range. T and NK cell numbers were normal. Responses to phytohemagglutinin and concanavalin A were normal, indicating normal T cell function. Neutrophil respiratory burst assays were normal. At the age of 1 year, immunologic re‐evaluation showed hypogammaglobulinemia‐IgG 142 mg/dL (5‐12 month reference range: 172‐1069 mg/dL), IgA < 10 mg/dL (7‐12 month reference range: 10‐106 mg/dL), and IgM 16 mg/dl (10‐12 month reference range: 41‐173 mg/dL).^6^ IgD^−^CD27^+^ switched memory B cells in the total B cells was 2.2%. According to European consensus classification for CVID,^7^, ^8^ switched memory B cells less than 2% were defined “severely reduced”. So, we considered 2.2% switched memory B cells to be “reduced” and followed him up as we were unable to exclude CVID. Since his psychomotor development was delayed, G‐banding stain and fluorescence in situ hybridization (FISH) was performed and del (16) (p11.2p12) was found. He was placed on prophylactic sulfamethoxazole‐trimethoprim without immunoglobulin replacement therapy (Figure 1). He did not have recurrent infections, allergic disease nor autoimmune diseases throughout the course. IgG level was fluctuating between 4 to 5 years of age, but normalized after 5 years of age (95% range of IgG level in relation of 4‐5 years old: 463‐1236 mg/dL)^9^ (Figure 1). He had normal vaccine responses during the period of hypogammaglobulinemia as well as after the recovery of IgG. We made a clinical diagnosis of THI rather than CVID due to his clinical course. By definition, the diagnosis of THI can be made with certainty only in retrospect.

His height and weight remained age‐appropriate, but he was found to have intellectual disability.

## 3 DISCUSSION

When hypogammaglobulinemia is detected early in life, THI needs to be differentiated from more serious primary immunodeficiency disorders that present similarly with a picture of humoral deficiency, including CVID and X‐linked Agammaglobulinemia (XLA). A definitive diagnosis of THI is only possible upon normalization of IgG levels. Demonstration of a normal vaccine response also made secondary causes of hypogammaglobulinemia unlikely. Presence of B and T cells made XLA and severe combined immunodeficiency (SCID) unlikely, respectively. CVID is a phenotypic diagnosis with a polygenic inheritance in most cases, and hence genetic evaluation could potentially be valuable in this patient, although there is currently no known correlation between clinical presentation and specific gene defects.^10^ This patient underwent serial evaluation of IgG levels, which was critical for eventual diagnosis of THI, but whilst waiting for the IgG levels to normalize, genetic evaluation for primary immunodeficiency was also carried out, including FISH.

As this patient was found to have both THI and 16p11.2 deletion syndrome, we explored the possibility of an association between these 2 clinical entities. 16p11.2 deletion has been reported to be associated with other forms of primary immunodeficiency including SCID and CVID,^5^, ^11^ making the association between THI and 16p111.2 deletion tenable. Maggadottir et al identified 11 single nucleotide polymorphisms (SNPs) at the 16p11.2 locus associated with CVID.^5^ The most significant SNP is in the gene fused‐in‐sarcoma (FUS), with 4 other SNPs mapping to integrin CD11b (ITGAM). The gene FUS has an intrinsic role in the proliferative responses of B cells to specific mitogenic stimuli.^12^ ITGAM encodes integrin alfa (CD11b). Integrins are heterodimeric integral membrane proteins with key roles in adhesion and cell contact. CD11b forms the leukocyte‐specific heterodimer macrophage receptor 1/complement receptor 3 (Mac‐1/CR3). CR3 is expressed on mononuclear phagocytes, neutrophils, natural killer cells, a subset of B cells,^13^ and some T cells. Thus, the ITGAM variant might impair these critical functions of the immune system. Unfortunately, these immunological and molecular analyses have not been done due to technical restrictions in our institutions.

Nevertheless, this case report may serve to add on to the spectrum of immunodeficiencies that may be associated with 16p11.2 deletion.

However, it is tenuous to ascribe a direct causal relationship between THI and 16p11.2 deletion for multiple reasons. Firstly, 16p11 deletion has been found to be the second most common deletion in patients who undergo chromosomal microarray (CMA)^4^ and patients with rare clinical conditions who undergo a CMA as a part of the evaluation may be found to have this genetic variation just due to the sheer frequently of this abnormality. In addition, to the author's knowledge, there is no other similar report of the same patient having both clinical entities of THI and 16p11.2 deletion, and there are numerous reports of patients with 16p11.2 deletion who do not have THI.

Although the underlying etiology of THI remains unclear and it is not known whether switched memory B cells are definitively reduced in THI, our data suggest that this phenomenon to be a possibility in THI. We suggest that analysis of SNPs at the 16p11.2 locus in future cases of THI may be of value in deepening the understanding of the underlying genetic bases.

## CONFLICT OF INTEREST

None declared.

## AUTHOR CONTRIBUTIONS

TI: participated in patient management, data collection and writing the draft, and critically reviewed the manuscript. SI: critically reviewed the manuscript. MH: critically reviewed the manuscript. YY: critically reviewed the manuscript. EA: contributed to the interpretation of the cases and critically reviewed the manuscript.
